# *JAK2^V617F^* Mutation Promoted IL-6 Production and Glycolysis *via* Mediating PKM1 Stabilization in Macrophages

**DOI:** 10.3389/fimmu.2020.589048

**Published:** 2021-02-08

**Authors:** Rongqing Li, Na Sun, Xin Chen, Xueqin Li, Jie Zhao, Wanpeng Cheng, Hui Hua, Masahiko Fukatsu, Hirotaka Mori, Hiroshi Takahashi, Hiroshi Ohkawara, Miwa Fukami, Masatoshi Okamoto, Yoichi Hamazaki, Kuiyang Zheng, Jing Yang, Takayuki Ikezoe

**Affiliations:** ^1^ Jiangsu Province Key Laboratory of Immunity and Metabolism, Xuzhou Medical University, Xuzhou, China; ^2^ Department of Pathogenic Biology and Immunology, Xuzhou Medical University, Xuzhou, China; ^3^ The Department of Hematology, Fukushima Medical University, Fukushima, Japan; ^4^ Department of Hematology, YUASA Foundation Jusendo General Hospital, Koriyama, Japan; ^5^ Department of Hematology, Iwaki City Medical Center, Iwaki, Japan

**Keywords:** JAK2V617F, glycolysis, PKM1, STAT3, IL-6

## Abstract

A substitution mutation of valine to phenylalanine at codon encoding position 617 of the Janus kinase 2 (*JAK2*) gene (*JAK2^V617F^*) has been detected in myeloid cells of some individuals with higher levels of proinflammatory cytokine production such as interleukin (IL)-6. However, the mechanisms by which *JAK2^V617F^* mutation mediating those cytokines remain unclear. We, therefore, established *JAK2^V617F^*-expressing murine macrophages (*JAK2^V617F^* macrophages) and found that the levels of p-STAT3 were markedly elevated in *JAK2^V617F^* macrophages in association with an increase in IL-6 production. However, inhibition of STAT3 by C188-9 significantly decreased the production of IL-6. Furthermore, the *JAK2^V617F^* mutation endowed macrophages with an elevated glycolytic phenotype in parallel with aberrant expression of PKM1. Interestingly, silencing of PKM1 inactivated STAT3 in parallel with reduced IL-6 production. In contrast, ectopic expression of PKM1 elevated IL-6 production *via* STAT3 activation. Importantly, the *JAK2^V617F^* mutation contributed to PKM1 protein stabilization *via* blockade of lysosomal-dependent degradation *via* chaperone-mediated autophagy (CMA), indicating that the *JAK2^V617F^* mutation could protect PKM1 from CMA-mediated degradation, leading to activation of STAT3 and promoting IL-6 production.

## Introduction

JAK2 is a non-receptor tyrosine kinase that activates signal transduction and activator of transcription (STAT) and mediates cytokine signaling. For example, erythropoietin binds to its receptor which in turn phosphorylates JAK2/STAT3 and regulates erythropoiesis (1). The *JAK2^V617F^* mutation was identified in the majority of individuals with polycythemia vera (PV) and more than 50% of those with essential thrombocythemia (ET) and primary myelofibrosis (2). *JAK2^V617F^* mutation confers ligand-independent activation of STATs (3). Additionally, transplantation of *JAK2^V617F^* -expressing hematopoietic cells resulted in erythrocytosis in a murine model (4).

The clinical features of PV and ET include thromboembolism, which limits the survival of these patients. For example, the *JAK2^V617F^* mutation was detected in individuals who developed Budd-Chiari syndrome, a potentially lethal thrombotic disease, even though they did not have any thrombotic risk factors or complete blood cell count abnormalities (5). Also, the *JAK2^V617F^* mutation was found in some individuals with age-related hematopoiesis, which is intimately linked to a high incidence of cardiovascular events (5). Moreover, veno-occlusive disease was more frequently noted in individuals with myelodysplastic syndromes harboring the *JAK2^V617F^* mutation than in those without the mutation (6).

Previous studies showed the aberrant production of cytokines in patients with myeloproliferative neoplasms (MPN). For example, multiplex cytokine analyses examined the serum levels of a variety of cytokines and chemokines in more than 400 patients with MPN and found the aberrant production of epidermal growth factor and GRO-α in these patients (7). Importantly, these chemokines were produced by CD56^+^CD14^+^ proinflammatory monocytes and their serum levels were predictable of disease progression (7). Intriguingly, monocytes isolated from MPN patients were insensitive to IL-10, a negative regulator of pro-inflammatory cytokines, resulting in the sustained production of tumor necrosis factor-α (8). Another study found that monocytes with *JAK2^V617F^* expressed a greater amount of CD25, an activate marker of monocytes than those with wild-type *JAK2*. Also, monocytes isolated from MPN patients with thrombotic history expressed a greater amount of CD25 compared with those isolated from MPN patients without thrombotic history (9). Collectively these results suggest the possible roles of cytokine produced by monocytes in the development of thrombosis in MPN patients.

A large number of studies suggest that the relationship between activated cytokine signaling, including that of interleukin (IL)-1β and IL-6, which are predominantly produced by macrophages, is involved in the development of atherothrombosis (10–12). In response to stimuli, the main macrophage functions are under metabolic control (13). Upon treatment with lipopolysaccharide (LPS), a specific Toll-like receptor 4 (TLR4) ligand, macrophages undergo metabolic reprogramming, leading to increased glycolysis and a disrupted Krebs cycle to supply cell metabolic adaptations and cytokine production (13). Pyruvate kinase M2 (PKM2), an indispensable member of the pyruvate kinase family, has been shown to influence glycolytic reprogramming in activated immune cells and tumor cells and to function as a proinflammatory mediator (14, 15). Upon LPS stimulation, PKM2 interacts with hypoxia-inducible factor 1-α (HIF-1α) in macrophages (16, 17). Additionally, PKM2 can be shuttled into nuclei and phosphorylate STAT3 (18), leading to an increase in IL-6 production (16). These observations prompted us to hypothesize that *JAK2^V617F^*-expressing cells, especially macrophages, produce large amounts of cytokines, which could play a role in the development of thrombosis in individuals possessing this mutation. To test this hypothesis, we established *JAK2^V617F^*-expressing macrophages and explored their function.

## Materials and Methods

### Cells

Murine RAW264.7 and J774A.1 cells were commercially obtained from FuHeng (FuHeng Cell Center, Shanghai, China). These cells were maintained in Dulbecco’s modified Eagle’s medium (DMEM) containing 10% heat-inactivated FBS, 100 mg/L streptomycins, and 100 U/ml penicillin at 37°C in a 5% CO_2_ environment.

### Gene Transfection and Silencing

For small interfering RNA (siRNA) transfections, cells were transfected with 20 nmol/L siRNA (Jima, Shanghai, China) by jetPRIME transfection reagent (Polyplus, France). For plasmid transfections, cells were transfected using jetPRIME (Polyplus, France) according to the manufacturer’s instructions. The siRNA sequences were as follows: siPKM1: GUGGAGGCCUCUUAUAAGUTT, siPPKM1#2 (the siRNA against PKM1 and PKM2): GCCACAGAAAGCUUUGCAUTT, siHSC70: CCAGGCCAGUAUUGAGAUUTT, and siLAMP2: GCCGUUCAGUCCAAUGCAUTT.

### Plasmids and Virus Infection

Human *JAK2* cDNA was synthesized and cloned into the pLenti-CMV-GFP-2A-Puro lentivirus vector (Abm, Canada) at NheI/XbaI sites. The *JAK2^V617F^* mutation was cloned using a TransStart FastPfu Fly DNA Polymerase Kit (TransGen Biotech). Lentiviruses were generated by cotransfecting 293T cells with the other two packing vectors pMD2G and psPAX and concentrated as described previously (19). Stable cell lines were generated by infection with lentivirus and selected with 2 mg/ml puromycin (Vicmed, Xuzhou, Jiangsu, China) for approximately 4 weeks. Murine *pkm1* cDNA (NM_001253883.1) was synthesized and cloned into the pLenti-GIII-CMV-CBH-GFP-2A-Puro vector (Abcam, Canada).

### Colony Formation Assay

For the colony formation assay, 200 infected cells were cultured in six-well plates at 37°C for 7–10 days, and visible colonies were washed twice with PBS, fixed with 4% paraformaldehyde, and stained with crystal violet. Pictures of the colonies were taken, and the number of colonies was counted by ImageJ software.

### RNA Isolation and Real-Time Reverse Transcription-Polymerase Chain Reaction

We measured the expression of actin for normalization. Real-time RT-PCR was performed using TB Green PCR Master Mix (TaKaRa, Japan). The primer sets for the PCR are shown in **Table 1**. The PCR conditions for all the genes were as follows: initial activation at 95°C for 30 s, followed by 40 cycles at 95°C for 5 s and at 60°C for 20 s, and fluorescence determination was performed at the melting temperature of the product for 15 s on a LightCycler 480 (Roche).

**Table 1:** Real-time RT-PCR primers.

Gene	Direction	Primer
*Il1b*	Forward	5’-CAGGCAGGCAGTATCACTCA-3’
	Reverse	5’-TGTCCTCATCCTGGAAGGTC-3’
*Il6*	Forward	5’-CCGGAGAGGAGACTTCACAG-3’
	Reverse	5’-TCCACGATTTCCCAGAGAAC-3’
*Hk1*	Forward	5’-CCAAAATAGACGAGGCCGTA-3’
	Reverse	5’-TTCAGCAGCTTGACCACATC-3’
*Pkm1*	Forward	5’-CCACTTGCAGCTATTCGAGG-3’
	Reverse	5’-CTGCAGCACTTGAAGGAGG-3’
*actin*	Forward	5’-GCTACAGCTTCACCACCACA-3’
	Reverse	5’-TCTCCAGGGAGGAAGAGGAT-3’

### Determination of mRNA Half-Life

To measure the half-life of endogenous HK1 or PKM1 messenger RNA (mRNA), cells were cultured in the presence of either control diluent or actinomycin D (2 µg/ml). Total RNA was extracted at the indicated time points and subjected to real-time RT-PCR. mRNA levels were normalized to actin and plotted as a percentage of the value at time 0 (set at 100%).

### Immunoblotting

Immunoblotting was performed as previously described (20). For examination of the protein involved in pro-survival signaling pathway, cell lysis from empty-, *JAK2*-, or *JAK2^V617F^*-expressing macrophages were extracted and subpackaged equally for three groups (each group included empty, *JAK2* and *JAK2^V617F^* lysates), followed by separation on 8% sodium dodecyl sulfate polyacrylamide gel electrophoresis (SDS-PAGE) gel. After semi-dry transfer, the membranes were sequentially probed with the indicated antibodies. Anti-p-JAK2 (T1007/1008) (#3776), anti-JAK2 (D2E12) (#3230), anti-p-STAT1 (T701) (#7649), anti-STAT1 (D1K9Y) (#14994), anti-p-STAT3 (Tyr705, D3A7) (#9145), anti-STAT3 (D3Z2G) (#12640), anti-p-STAT5 (Tyr694, C11C5) (#9359), anti-STAT5 (D206Y) -(#94205), anti-p-p38 (Thr180/Tyr182, D3F9) (#4511), anti-p38 (D13E1) (#8690), anti-p-AKT (Ser473, D9E) (#10019), anti-AKT (#9272), anti-p-JNK (Thr183/Tyr185, 81E11) (#4668), and anti-JNK (#9252) antibodies were purchased from Cell Signaling Technology. Anti-HK1 (19662-1-AP), anti-HK2 (22029-1-AP), anti-PKM1 (15821-1-AP), anti-flag (Sigma), and anti-β-actin (66009-1-Ig) antibodies were purchased from ProteinTech.

### Half-Life Assay

Cells were treated with 200 μg/ml cycloheximide and harvested using sodium dodecyl sulfate (SDS) lysis buffer at the indicated time points. The levels of HK2 and β-actin were analyzed by Western blotting. HK2 bands were quantified after normalization to those of β-actin, and the quantification was plotted as the relative amount of protein remaining compared to that at the 0 min treatment time. The bands were compared quantitatively using ImageJ software.

### Immunoprecipitation

Immunoprecipitation was performed as described previously (21). Briefly, RAW264.7 lysates were prepared in immunoprecipitation lysis buffer (20 mmol/L Tris-Cl, pH 8.0, 10 mmol/L NaCl, 1 mmol/L EDTA, 0.1% NP-40) containing a protease inhibitor cocktail (Sigma). Two micrograms of cell extracts were precleared with 50 µl of protein A/G-agarose (Thermo Fisher) at 4°C for 2 h, and the supernatant was incubated with the corresponding antibodies with gentle shaking at 4°C overnight, followed by the addition of 20 µl of protein A/G-agarose for another 1 h. The beads were washed and then resuspended in 30 µl of loading buffer and boiled for 5 min, followed by Western blot detection.

### Seahorse Analyzer

The extracellular acidification rate (ECAR) was measured with an XF 24 extracellular flux analyzer (Seahorse Bioscience). Briefly, 8x10^4^ cells were seeded in each well of Seahorse XF 24 plates with 250 μl of DMEM and incubated overnight. ECAR measurements were normalized to the cell number. Cells were initially plated in XF Seahorse media with 2 mM glutamine in ECAR tests using the following concentrations of injected compounds, as indicated in the text: oligomycin, 2 µM; 2-DG, 100 mM; and glucose, 30 mM.

### Glucose Uptake Assay

Glucose uptake was examined by a Glucose Assay Kit (Sigma) according to the manufacturer’s instructions.

### Measurement of Serum Levels of IL-6 in MPN Patients

Serum was drawn from patients with PV or ET after obtaining written informed consent. Levels of IL-6 were measured using a human IL-6 ELISA kit (ProteinTech, Rosemont, IL, USA). This study was approved by the ethical committee of Fukushima Medical University.

### Statistical Analysis

Data are expressed as means ± SEMs. Data shown are representative of one of two or three independent experiments. The comparison was performed by GraphPad Prism V5 (GraphPad Software). Mann-Whitney U test was used to analyze the comparison of IL-6 concentration in MPN patients with/without the *JAK2^V617F^* mutation. The student’s t-test was used to analyze the comparison of IL-6 concentrations in the knockdown or overexpression system. One-way analysis of variance (ANOVA) was used to analyze the comparison of empty-, *JAK2*-, or *JAK2^V617F^*-expressing macrophages. Upon stimuli, two-way ANOVA was used for comparison of empty-, *JAK2*-, or *JAK2^V617F^*-expressing macrophages.

## Results

### *JAK2^V617F^* Mutation Confers STAT Activation and Production of IL-6

**Figure 1:**
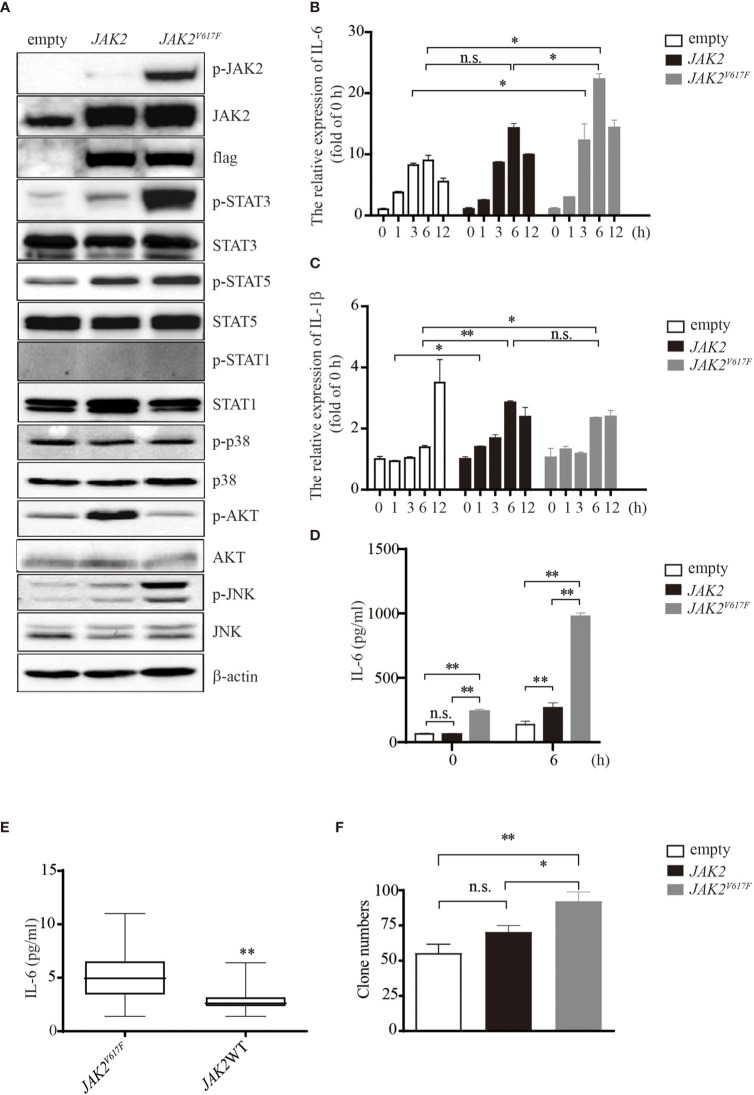
IL-6 production by *JAK2^V617F^*-expressing cells *in vitro* and *in vivo*. **(A)** Western blot analysis. Proteins were extracted from the indicated cell lines and sub packaged equally for three groups (each group included the dame amount of empty, *JAK2* and *JAK2^V617F^* lysates), followed by separation on 8% sodium dodecyl sulfate polyacrylamide gel electrophoresis (SDS-PAGE) gel. The membrane was sequentially probed with the indicated antibodies. The results shown are representative of one of two independent experiments. **(B)** The relative expression of *Il6* in cells treated with or without 100 ng/ml lipopolysaccharide (LPS) for the indicated times. The data shown are representative of one of three independent experiments performed in duplicate. The results represent the mean ± SEM. Two-way analysis of ANOVA was used for comparison. *P < 0.05. **(C)** The relative expression of *Il1b* in cells treated with or without 100 ng/ml LPS for the indicated times. The data shown are representative of one of three independent experiments performed in duplicate. The results represent the mean ± SEM. Two-way analysis of ANOVA was used for comparison. **P < 0.01; *P < 0.05; n.s., not significant. **(D)** The IL-6 concentration of the cell culture medium was detected by ELISA. The data shown are representative of one of two independent experiments performed in duplicate. The results represent the mean ± SEM. Two-way analysis of ANOVA was used for comparison. **P < 0.01; n.s., not significant. **(E)** Measurement of IL-6 in MPN patients with/without the *JAK2^V617F^* mutation. Blood was withdrawn from patients, and the levels of IL-6 were measured by ELISA. The data shown are representative of one of two independent experiments. The results represent the mean ± SEM. Statistical significance was assessed by the Mann-Whitney U test. **, *P* < 0.01. **(F)** Colony assay. The number of colonies was counted by ImageJ software after crystal violet staining. The data shown are representative of three independent experiments performed in duplicate. The results represent the mean ± SEM. One-way analysis of ANOVA was used for comparison. **P < 0.01; *P < 0.05; n.s., not significant.

*JAK2^V617F^* mutation is associated with the development of thrombosis, in which cytokines may be key orchestrators. To investigate whether and how *JAK2^V617F^ -*expressing macrophages can produce a large amount of cytokines, such as IL-1β and IL-6, murine macrophage RAW264.7 cells were transduced with either an empty vector or the *JAK2* or *JAK2^V617F^* gene by using lentiviruses, and sublines stably expressing these genes were established (**Figure 1A**). *JAK2^V617F^* macrophages aberrantly expressed the phosphorylated forms of JAK2 (p-JAK2) and STAT3 (p-STAT3) (**Figure 1A**). Additionally, the levels of p-JNK were also dramatically increased in *JAK2^V617F^* macrophages (**Figure 1A**). In contrast, the levels of p-STAT5 were modestly increased in *JAK2^V617F^* macrophages (**Figure 1A**). Interestingly, p-STAT1, the key mediator of interferon signaling involved in thrombus resolution (22), was undetectable in *JAK2* and *JAK2^V617F^* macrophages (**Figure 1A**). Unexpectedly, the levels of p-AKT were decreased in *JAK2^V617F^* macrophages compared to those in empty vector-transduced macrophages, although the levels of p-AKT were elevated in *JAK2* -expressing macrophages (**Figure 1A**). Similarly, *JAK2^V617F^* mutation endowed murine J774A.1 macrophages with higher levels of p-STAT3, but not p-STAT1, p-p38, p-AKT, or p-JNK, compared with that in JAK2-expressing J774A.1 macrophages (**Figure S1A**). The levels of p-STAT5 were slightly increased in *JAK2^V617F^*-expressing J774A.1 macrophages, compared with that in JAK2-expressing J774A.1 macrophages (**Figure S1A**).

Exposure of macrophages to LPS increased the expression levels of IL-6 in a time-dependent manner up to 6 h (**Figure 1B**). *JAK2^V617F^* macrophages expressed greater amounts of IL-6 mRNA than empty vector- or *JAK2* expression vector-transduced RAW264.7 macrophages (empty or JAK2 macrophages) after exposure to LPS (**Figure 1B**). On the other hand, the levels of IL-1β mRNA were lower in *JAK2^V617F^* macrophages at 12 h after exposure to LPS than in empty macrophages (**Figure 1C**). The levels of IL-1β mRNA after exposure to LPS were comparable between *JAK2^V617F^* and *JAK2* macrophages (**Figure 1C**). An ELISA showed that the production of IL-6 by *JAK2^V617F^* macrophages was greater than that by empty and *JAK2* macrophages at baseline and after exposure to LPS for 6 h (**Figure 1D**).

We next measured the serum levels of IL-6 in MPN patients with/without the *JAK2^V617F^* mutation. Importantly, an increase in the levels of IL-6 was noted in PV and ET patients harboring *JAK2^V617F^* compared with those in patients without *JAK2^V617F^* (**Figure 1E**). White blood cells (WBCs) and values of C-reactive proteins (CRPs) were comparable between the two groups (**Table 2**). Of note, five thrombotic events were noted in MPN patients with *JAK2^V617F^* but not in patients without *JAK2* mutations (**Table 2**).

**Table 2:** Clinical characteristics of patients with polycythemia vera (PV) or thrombocythemia (ET).

	JAK2V617F (n=24)	JAK2 WT (n=12)	p value
Diagnosis	PV n=12	PV n=6	
	ET n=12	ET n=6	
CALR mutation	None	n=2	
Thrombotic events	Cerebral infarction n=3	None	
	Deep vein thrombosis n=1		
	Portal vein thrombosis n=1		
IL-6 (pg/mL)	5.1 (2.7–11)	3.0 (1.4–6.4)	0.0022
WBC (x103/µL)	15.2 (3.3–20.9)	8.1 (3.8–20.2)	0.16
Hemoglobin (g/dL)	14 (11.1–19.4)	15.1 (7.9–19.6)	0.21
Platelets (x104/µ L)	53.2 (11.3–97.7)	54.8 (3.2–134)	0.67
CRP (mg/dL)	0.5 (0.01–2.15)	0.2 (0.02–1.09)	0.88

WT, wild-type; CALR, calreticulin; IL-6, interleukin-6; WBC, white blood cell count; CRP, C-reactive protein; PV, polycythemia vera; ET, essential thrombocytosis. Statistical analysis was performed by the Mann-Whitney U test.

Because the JAK/STAT pathway is involved in cell proliferation, we performed a colony assay and found that *JAK2^V617F^* macrophages formed more colonies than empty and *JAK2* macrophages (**Figures 1F** and **S1B**). All of these observations suggested that the *JAK2^V617F^* mutation endowed macrophages with activation of STAT along with abundant production of IL-6.

### Activation of STAT3 Was Required for IL-6 Production in *JAK2^V617F^* Macrophages

**Figure 2:**
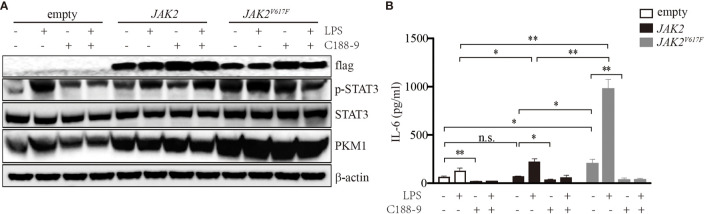
Activation of STAT3 mediated by the *JAK2^V617F^* mutation was critical for IL-6 production. **(A)** Western blot analysis. Cells were cultured with 100 ng/ml LPS and/or 5 µM C188-9, and proteins were extracted and subjected to Western blot analysis. The membrane was sequentially probed with the indicated antibodies. The results shown are representative of one of two independent experiments. **(B)** The IL-6 concentration of the cell culture medium was detected by ELISA. The data shown are representative of one of two independent experiments performed in duplicate. The results represent the mean ± SEM. Two-way analysis of ANOVA was used for comparison. **P < 0.01; *P < 0.0; n.s., not significant.

To determine whether STATs were critical for IL-6 production in *JAK2^V617F^* macrophages, we treated these cells with the STAT3 inhibitor C188-9. As shown in **Figure 2A**, upon LPS treatment, the levels of p-STAT3 were dramatically increased. By contrast, the levels of p-STAT3 were inhibited in the presence of C188-9 (**Figure 2A**). As expected, inhibition of STAT3 attenuated the IL-6 production induced by LPS (**Figure 2B**). Since STAT5 and JNK were activated in the presence of the *JAK2^V617F^* mutation, to test whether STAT5 and JNK were also involved in mediating IL-6 production in *JAK2^V617F^* macrophages, we blocked the STAT5 and JNK signal transduction pathways by specific inhibitors. We found that the inhibition of these pathways did not modulate the levels of IL-6 (data not shown). These observations suggested that activation of STAT3 was critical for IL-6 production.

### *JAK2^V617F^* Mutation Increases Glycolysis in Association with Constitutive Expression of PKM1 in Macrophages

**Figure 3:**
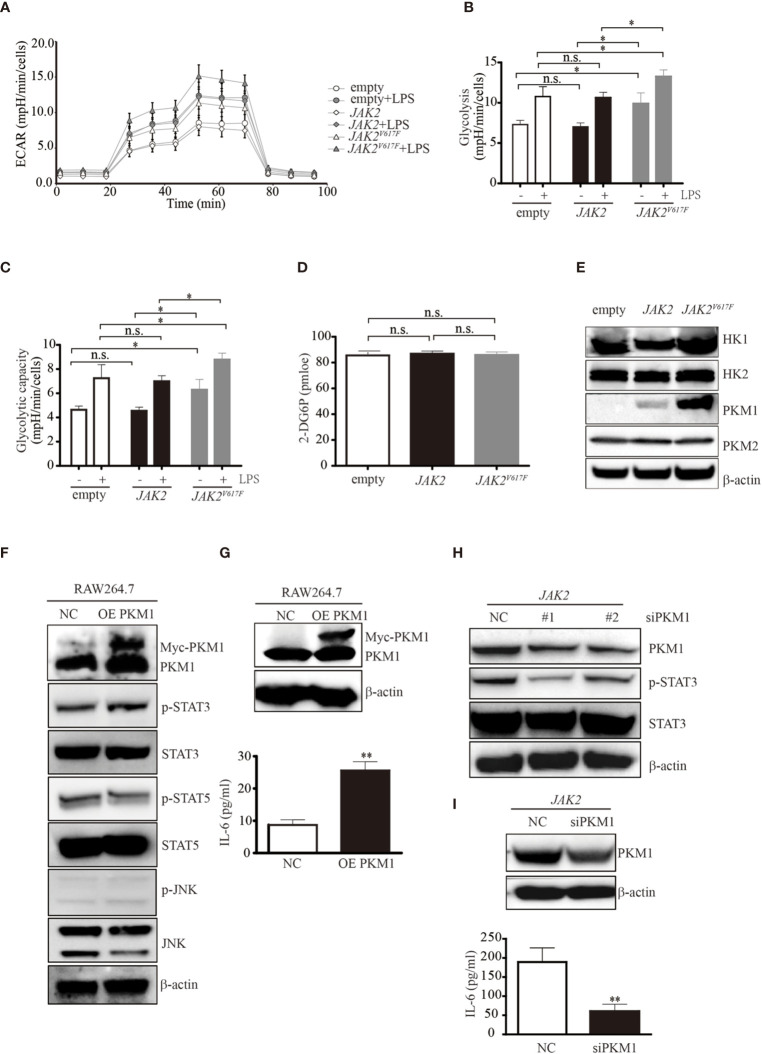
The *JAK2^V617F^* mutation endowed cells with an increase in glycolysis. **(A)** Cells treated with or without LPS were seeded into wells, and the ECAR was determined by extracellular flux analysis. A representative plot of the ECAR over time of these cells with the addition of glucose (30 mM), oligomycin (2 µM), and 2-DG (50 mM), as indicated. **(B)** Glycolysis in the assay shown in **Figure 3A** was quantified. The data shown are representative of one of two independent experiments performed in triplicate. The results represent the mean ± SEM. Two-way analysis of ANOVA was used for comparison. *P < 0.05. **(C)** The glycolytic capacity in the assay shown in **Figure 3A** was quantified. The data shown are representative of one of two independent experiments performed in triplicate. The results represent the mean ± SEM. Two-way analysis of ANOVA was used for comparison. *P < 0.05. **(D)** The levels of glucose uptake in the indicated macrophages. The data shown are representative of one of two independent experiments. The results represent the mean ± SEM. One-way analysis of ANOVA was used for comparison. n.s., not significant. **(E)** Western blot analysis. Proteins were extracted and subjected to Western blot analysis. The membrane was sequentially probed with the indicated antibodies. The results shown are representative of one of two independent experiments. **(F)** Western blot analysis. RAW264.7 cells were transfected with empty or Myc-PKM1 expression vectors. After 24 h, proteins were extracted and subjected to Western blot analysis. The membrane was sequentially probed with the indicated antibodies. The results shown are representative of one of two independent experiments. **(G)** The IL-6 concentration of the cell culture medium was detected by ELISA. The data shown are representative of one of two independent experiments performed in triplicate. The results represent the mean ± SEM. Two-way analysis of ANOVA was used for comparison. **P < 0.01. **(H)** Western blot analysis. *JAK2*-expressing RAW264.7 cells were transfected with a negative control siRNA or an siRNA against PKM1. After 24 h, proteins were extracted and subjected to Western blot analysis. The membrane was sequentially probed with the indicated antibodies. The results shown are representative of one of two independent experiments. **(I)** The IL-6 concentration of the cell culture medium was detected by ELISA. The data shown are representative of one of two independent experiments performed in triplicate. The results represent the mean ± SEM. Two-way analysis of ANOVA was used for comparison. **P < 0.01.

In response to stimulation, macrophages undergo dynamic metabolic reprogramming (23). Activation of macrophages upon stimulation by LPS occurs in association with a metabolic switch from oxidative phosphorylation to glycolysis (24). To investigate whether an elevated glycolytic phenotype occurs in *JAK2^V617F^* macrophages, we compared the glycolytic capacities among empty, *JAK2*, and *JAK2^V617F^* macrophages. A Seahorse analyzer measured the ECAR in macrophages after sequential addition of glucose, oligomycin, and 2-DG (**Figures 3A** and **S2A**). Both glycolysis and glycolytic capacity were increased in *JAK2^V617F^* macrophages compared to those in empty and *JAK2* macrophages, and these capacities were further enhanced in the presence of LPS (**Figures 3B, C**). Similarly, both glycolysis and glycolytic capacity were increased in *JAK2^V617F^*-expressing J774A.1 cells compared to those in empty-expressing J774A.1 cells, and these capacities were further enhanced in the presence of LPS (**Figures S2B, C**). Additionally, there’s a tendence that both glycolysis and glycolytic capacity were increased in *JAK2^V617F^*-expressing J774A.1 macrophages compared to those in JAK2-expressing J774A.1 cells, which were further enhanced in the presence of LPS (**Figures S2B, C**). On the other hand, glucose intake was almost identical in each subline (**Figure 3D**).

To explore the underlying mechanisms causing the elevated glycolytic phenotype noted in *JAK2^V617F^* macrophages, we next measured the expression levels of enzymes involved in glycolysis and found that pyruvate kinase M 1 (PKM1) but not hexokinase 2 (HK2) or PKM2 was aberrantly expressed in *JAK2^V617F^* macrophages compared to those in the other two sublines (**Figure 3E**). HK1 was slightly induced in *JAK2^V617F^* macrophages compared to in the other two sublines (**Figure 3E**). As expected, the expression of PKM1, but not HK1/2 or PKM2 was dramatically increased in *JAK2^V617F^*-expressing J774A.1 macrophages compared to those in the other two sublines (**Figure S2D**).

### *JAK2^V617F^* Mutation-Induced Phosphorylation of STAT3 by PKM1

A previous study showed that PKM2 promotes proinflammatory cytokine production by mediating the activation of STAT3 (25). To further investigate the role of elevated PKM1 observed in *JAK2^V617F^* macrophages, we transfected RAW264.7 macrophages with PKM1 plasmid DNA and found that overexpression of PKM1 caused an increase in IL-6 production (**Figures 3F, G**). Additionally, overexpression of PKM1 increased the levels of p-STAT3 but not those of p-STAT5 and p-JNK (**Figure 3F**). Silencing of PKM1 decreased the levels of p-STAT3 (**Figure 3H**) in parallel with a decrease in IL-6 production (**Figure 3I**). Similarly, ectopic expression of PKM1 in J774A.1 macrophages contributed to higher levels of p-STAT3 in parallel with an increase in IL-6 production (**Figures S2E, F**). Whereas, knocking down of PKM1 reduced the expression of p-STAT3 along with a decrease in IL-6 production (**Figures S2G, H**), suggesting that the *JAK2^V617F^* mutation could endow macrophages with elevated IL-6 production by PKM1-mediated activation of STAT3.

### *JAK2^V617F^* Mutation Contributed to PKM1 Protein Stabilization

**Figure 4:**
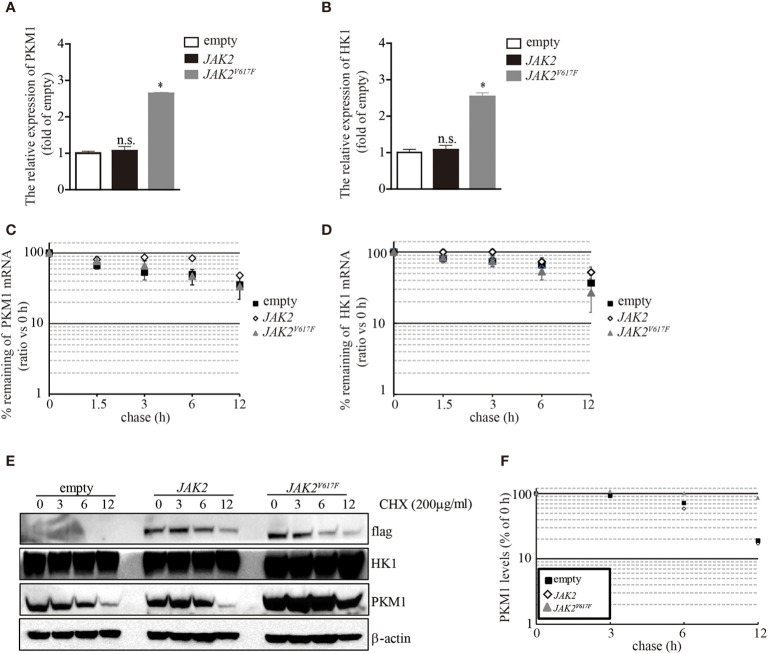
The *JAK2^V617F^* mutation facilitated PKM1 protein stabilization. **(A)** The relative expression of HK1 messenger RNA (mRNA) in empty vector-transfected RAW264.7, *JAK2*-expressing RAW264.7, or *JAK2^V617F^*-expressing RAW264.7 cells. The data shown are representative of one of three independent experiments performed in duplicate. The results represent the mean ± SEM. One-way analysis of ANOVA was used for comparison. *P < 0.05; n.s., not significant. **(B)** The relative expression of PKM1 mRNA in empty vector-transfected RAW264.7, *JAK2*-expressing RAW264.7, or *JAK2^V617F^*-expressing RAW264.7 cells. The data shown are representative of one of three independent experiments performed in duplicate. The results represent the mean ± SEM. One-way analysis of ANOVA was used for comparison. *P < 0.05; n.s., not significant. **(C)** Actinomycin D was added for the indicated period to block RNA synthesis, and HK1 mRNA was analyzed using real-time RT-PCR. The data shown are representative of one of two independent experiments performed in duplicate. Two-way analysis of ANOVA was used for comparison. **(D)** Actinomycin D was added for the indicated period to block RNA synthesis, and PKM1 mRNA was analyzed using real-time RT-PCR. The data shown are representative of one of two independent experiments performed in duplicate. Two-way analysis of ANOVA was used for comparison. **(E–F)** The levels of HK1, PKM1, and actin were analyzed by Western blotting. The amounts of HK1 and PKM1 in empty vector-transfected, *JAK2*-expressing, or *JAK2^V617F^*-expressing RAW264.7 cells were quantified by densitometry, normalized to the level of actin, and plotted. The data shown are representative of one of two independent experiments.

The aberrant expression of PKM1 in *JAK2^V617F^* macrophages prompted us to ask whether the *JAK2^V617F^* mutation induced an increase in *pkm1* gene expression. We found that in *JAK2^V617F^* macrophages, the level of PKM1 mRNA was increased by 2.5-fold compared to that in empty macrophages (**Figure 4A**). However, the level of PKM1 mRNA in *JAK2* macrophages was almost identical to that in empty macrophages (**Figure 4A**). Similarly, the level of HK1 mRNA was increased in *JAK2^V617F^* macrophages compared to that in empty or *JAK2* macrophages (**Figure 4B**). Next, actinomycin D was used to block *de novo* RNA synthesis (26), and the persistence of either PKM1 or HK1 mRNA was measured by real-time RT-PCR. The *JAK2^V617F^* mutation did not change PKM1 (**Figure 4C**) and HK1 mRNA degradation (**Figure 4D**), which excludes the possibility that the *JAK2^V617F^* mutation induced PKM1 expression *via* mainly stabilization of PKM1 mRNA. In addition, these results prompted us to investigate whether protein degradation was critical for *JAK2^V617F^* mutation-mediated PKM1 expression.

To elucidate whether the *JAK2^V617F^* mutation influences PKM1 protein degradation, we performed a protein half-life assay. In macrophages infected with an empty lentivirus or the JAK2 lentivirus, PKM1 had a modest half-life of approximately 6 h (**Figures 4E, F**). By contrast, in the presence of the *JAK2^V617F^* mutation, PKM1 degradation was hampered (**Figures 4E, F**). After 12 h of tracing, the protein levels of PKM1 were only slightly reduced in *JAK2^V617F^* macrophages (**Figures 4E, F**). However, HK1 was relatively stable, and its half-life appeared to be unaffected (**Figure 4E**). Together, these data strongly indicated that the *JAK2^V617F^* mutation played an important role in PKM1 protein degradation.

### The *JAK2^V617F^* Mutation Facilitated HSC70 Binding

**Figure 5:**
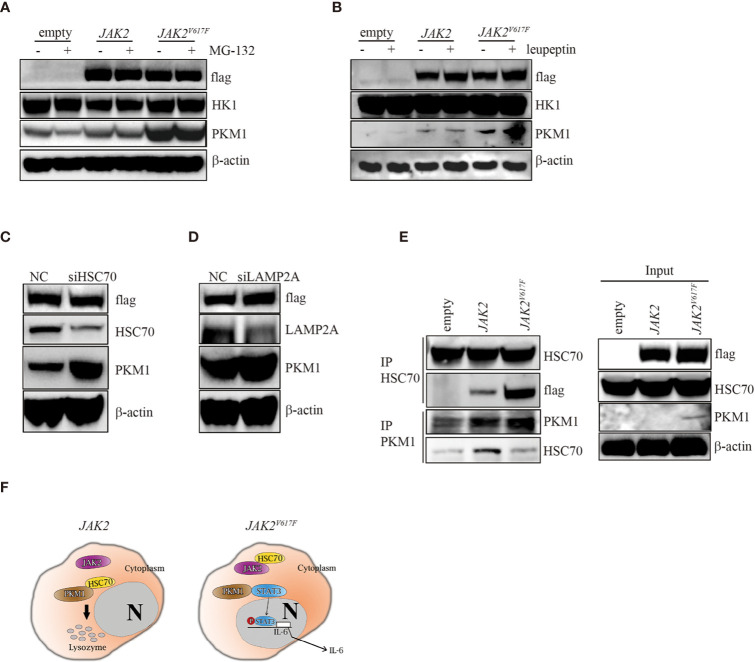
The *JAK2^V617F^* mutation protected PKM1 protein from CMA-mediated degradation. **(A)** Western blot analysis. Cells were cultured with 10 µM MG-152 for 5 h, and proteins were extracted and subjected to Western blot analysis. The membrane was sequentially probed with the indicated antibodies. The results shown are representative of one of two independent experiments. **(B)** Western blot analysis. Cells were cultured with 10 µM leupeptin for 18 h, and proteins were extracted and subjected to Western blot analysis. The membrane was sequentially probed with the indicated antibodies. The results shown are representative of one of three independent experiments. **(C)** Western blot analysis. *JAK2*-expressing RAW264.7 cells were transfected with a negative control siRNA or an siRNA against HSC70. After 24 h, proteins were extracted and subjected to Western blot analysis. The membrane was sequentially probed with the indicated antibodies. The results shown are representative of one of three independent experiments. **(D)** Western blot analysis. *JAK2*-expressing RAW264.7 cells were transfected with a negative control siRNA or an siRNA against LAMP2A. After 24 h, proteins were extracted and subjected to Western blot analysis. The membrane was sequentially probed with the indicated antibodies. The results shown are representative of one of three independent experiments. **(E)** IP. Cell lysates were harvested from empty vector-transfected, *JAK2*-expressing and *JAK2^V617F^*-expressing RAW264.7 cells, submitted to IP for either HSC70 or PKM1, and immunoblotted with the indicated antibodies. The loading represents 5% of the total cell lysate utilized for IP. The results shown are representative of one of three independent experiments. **(F)** Signaling pathways in normal and *JAK2^V617F^* cells. In normal cells, HSC70 interacts with PKM1 and promotes PKM1 degradation (left panel). The *JAK2^V617F^* mutation sequesters PKM1 from binding with HSC70, resulting in PKM1 accumulation, which causes upregulation of the transcriptional activity of STAT3 and subsequently leads to enhancement of IL-6 production (right panel).

In search of the mechanisms by which the *JAK2^V617F^* mutation inhibited PKM1 degradation, we treated the three macrophage sublines with either the proteasome inhibitor MG-132 or the lysosomal protease inhibitor leupeptin and found that upon MG-132 treatment, the levels of PKM1 were not modulated (**Figure 5A**). On the other hand, the levels of PKM1 were increased in the presence of leupeptin in *JAK2* and *JAK2^V617F^* macrophages (**Figure 5B**). A recent study indicated that acetylation of PKM2 on lysine promoted its lysosomal-dependent degradation *via* chaperone-mediated autophagy (CMA) (27), a pathway for selective degradation of individual proteins that is mediated through binding to heat shock cognate 71 kDa protein (HSC70) followed by unfolding and translocation of proteins through the lysosomal membrane by lysosome-associated membrane protein type 2A (LAMP2A) (28). Given that *JAK2^V617F^* macrophages possess higher levels of PKM1 (**Figures 2** **–** **4**) than empty macrophages, we hypothesized that lysosomal degradation of PKM1 occurred through CMA. To test this hypothesis, we transfected *JAK2* macrophages with siRNA against either HSC70 or LAMP2A. As expected, silencing of HSC70 or LAMP2A induced an increase in the levels of PKM1 in *JAK2^V617F^* macrophages (**Figures 5C, D**).

Since JAK2 interacts with HSC70 (29), we hypothesize that the JAK2^V617F^ mutant protein could be a preferred form for HSC70 binding compared with wild-type JAK2, protecting PKM1 from CMA-mediated degradation. To test this hypothesis, we performed an immunoprecipitation (IP) analysis. IP of HSC70 pulled down a significant amount of flag in *JAK2^V617F^* macrophage lysates (**Figure 5E**, IP lane 3). In comparison, the same amount of HSC70 pulled down less flag in *JAK2* macrophage lysates (**Figure 5E**, IP lane 2). Although it has been shown that HSC70 binds PKM2 (27), unfortunately, the IP of HSC70 did not pull down PKM1 *in vivo* (data not shown), perhaps because the anti-HSC70 antibody interfered with binding. IP of PKM1 pulled down a significant amount of HSC70 in *JAK2* macrophage lysates (**Figure 5E**, IP PKM1, lane 2). In comparison, a higher amount of PKM1 pulled down less HSC70 in *JAK2^V617F^* macrophage lysates (**Figure 5E**, IP PKM1, lane 3).

Together, these data demonstrated that the JAK2^V617F^ mutant protein bound HSC70 more efficiently than the wild-type JAK2 protein. They also suggested that the JAK2^V617F^ mutant protein could protect PKM1 from CMA-mediated degradation through abrogating HSC70-PKM1 binding.

## Discussion

Recent studies have demonstrated that the *JAK2^V617F^* mutation triggers the activation of STAT1 in myeloid cells. As a consequence, exposure of *JAK2^V617F^*-expressing myeloid cells to LPS produced greater amounts of IL-6 than that produced by control cells, which was consistent with the current study results (**Figure 1**). Interestingly, transplantation of *JAK2^V617F^*-harboring hematopoietic stem cells into mice accelerated cardiac hypertrophy and fibrosis, which were accompanied by a large number of infiltrating macrophages and elevated IL-6 expression in the heart (30). Another study also showed that the transplantation of *JAK2^V617F^*-expressing hematopoietic stem cells enhanced the formation of atherosclerotic lesions in lipoprotein receptor knockout mice. The authors also found that *JAK2^V617F^* macrophages produced large amounts of cytokines and chemokines (31). Collectively, these observations suggested that *JAK2^V617F^* macrophages acquire activated inflammatory properties and contribute to the development of cardiovascular diseases.

The present study found that forced expression of *JAK2^V617F^* resulted in an increase in the production of IL-6 but not IL-1β in macrophages even in the absence of LPS (**Figure 1D** and data not shown). Additionally, elevated serum levels of IL-6 were noted in MPN patients harboring *JAK2^V617F^* (**Figure 1E**, **Table 2**). To explore the mechanisms by which *JAK2^V617F^* mutant proteins cause aberrant expression of inflammatory cytokines in macrophages, we focused on metabolic reprogramming in these cells. Surprisingly, we found that *JAK2^V617F^* macrophages possessed increased levels of PKM1 in parallel with an increase in the glycolytic phenotype (**Figures 3** and **S2**). Previous studies showed that PKM2 phosphorylated STAT3 (18), contributing to elevated IL-6 production (16). Similar to the role of PKM2 in immune cells (25), PKM1 also activates STAT3. However, in contrast to PKM2, forced expression of PKM1 endowed macrophages with an increase in the levels of IL-6 but not IL-1β (**Figure 3**), suggesting that PKM1 specifically regulates the expression of IL-6. To support this idea, silencing of PKM1 hampered IL-6 production but not IL-1β production (data not shown) in association with a decrease in the levels of p-STAT3 (**Figure 3**). Furthermore, a specific inhibitor of STAT3 downregulated endogenous expression of IL-6 in *JAK2^V617F^* macrophages (**Figure 2**). These observations suggested that an enhanced glycolytic phenotype resulted in activation of STAT3, leading to elevated production of IL-6 mediated by *JAK2^V617F^*; aberrant expression of PKM1 mediated by *JAK2^V617F^* could play an important role in regulating this process in macrophages.

Unlike PKM2, PKM1 forms a stable, constitutively active tetramer with high PK activity and is aberrantly expressed in differentiated cells, such as those of the muscle and brain (32). Whether tetrameric PKM1 could be converted into a PKM1 dimer following posttranslational modifications remains obscure. Interestingly, in *JAK2^V617F^* macrophages, PKM1 protein half-life was prolonged compared to that in their parental cell lines (**Figures 4E, F**), although the levels of PKM1 mRNA were also increased in *JAK2^V617F^* macrophages (**Figure 4**), indicating that the JAK2^V617F^ protein could be involved in controlling PKM1 protein stabilization, at least partially. In a further study, we found that JAK2^V617F^ interacted with HSC70, releasing PKM1 from HSC70-mediated degradation, which in turn could enhance PKM1 protein stabilization (**Figure 5**).

Importantly, JAK2/STAT3 is not the only signaling that governs the cytokine production in macrophages. The use of ruxolitinib, an inhibitor of JAK2 was not able to decrease cytokine production in MPN patients. *Ex vivo* studies found that inhibition of nuclear factor κB and MAPK signaling is also required to shut down the production of cytokines in MPN patients (33).

Altogether, our results demonstrate that the *JAK2^V617F^* mutation stimulates IL-6 production through activation of STAT3 signaling, which is at least in part mediated by PKM1-regulated glycolysis. Targeting the PKM1/STAT3/IL-6 axis may be a promising treatment strategy to prevent thrombosis and cardiovascular events in individuals harboring the *JAK2^V617F^* mutation.

## Data Availability Statement

The datasets presented in this study can be found in online repositories. The names of the repository/repositories and accession number(s) can be found in the article/**Supplementary Material**.

## Ethics Statement

Written informed consent was obtained from the individual(s) for the publication of any potentially identifiable images or data included in this article.

## Author Contributions

Conception and design: JY and TI. Development of methodology: RL, NS, and JY. Acquisition of data (provided animals, enrolled and managed patients, provided facilities, etc.): TI. Analysis and interpretation of data (e.g., statistical analysis, biostatistics, and computational analysis): JY and TI. Writing, review, and/or revision of the manuscript: JY and TI. Administrative, technical, or material support (i.e., reporting or organizing data and constructing databases): RL, NS, XC, XL, JZ, HH, WC, MFukat, HM, HT, HO, MFukam, MO, YH, KZ. Study supervision: TI. All authors contributed to the article and approved the submitted version.

## Funding

This work was supported by grants from the Jiangsu Distinguished Professorship Program to JY and KAKENHI (18H02844) to TI.

## Conflict of Interest

The authors declare that the research was conducted in the absence of any commercial or financial relationships that could be construed as a potential conflict of interest.

## Supplementary Material

Supplementary Figure 1 Related to **Figure 1**. **(A)** Western blot analysis. Proteins were extracted from the indicated cell lines and sub packaged equally for three groups (each group included the same amount of empty, JAK2 and JAK2V617F lysates), followed by separation on 8% SDS-PAGE gel. The membrane was sequentially probed with the indicated antibodies. The results shown are representative of one of two independent experiments. **(B)** Colony assay. The number of colonies was counted by ImageJ software after crystal violet staining. The data shown are representative of three independent experiments performed in duplicate. The results represent the mean ± SEM. One-way analysis of ANOVA was used for comparison. ***P* < 0.01; **P* < 0.05; n.s., not significant.

Supplementary Figure 2 Related to **Figures 3**. **(A)** J774A.1 macrophages treated with or without LPS were seeded into wells, and the ECAR was determined by extracellular flux analysis. A representative plot of the ECAR over time of these cells with the addition of glucose (30 mM), oligomycin (2 mM), and 2-DG (50 mM), as indicated. **(B)** Glycolysis in the assay shown in **Supplementary Figure 2A** was quantified. The data shown are representative of one of two independent experiments. The results represent the mean ± SEM. Two-way analysis of ANOVA was used for comparison. **P* < 0.05. **(C)** The glycolytic capacity in the assay shown in **Figures 3A** was quantified. The data shown are representative of one of two independent experiments. The results represent the mean ± SEM. Two-way analysis of ANOVA was used for comparison. **P* < 0.05. **(D)** Proteins were extracted and subjected to Western blot analysis. The membrane was sequentially probed with the indicated antibodies. The results shown are representative of one of two independent experiments. **(E)** J774A.1 macrophages were transfected with empty or Myc-PKM1 expression vectors. After 24 hrs, proteins were extracted and subjected to Western blot analysis. The membrane was sequentially probed with the indicated antibodies. The results shown are representative of one of two independent experiments. **(F)** The IL-6 concentration of the cell culture medium was detected by ELISA. The data shown are representative of one of two independent experiments performed in triplicate. The results represent the mean ± SEM. Two-way analysis of ANOVA was used for comparison. ***P* < 0.01. **(G)** JAK2-expressing J774A.1 cells were transfected with a negative control siRNA or a siRNA against PKM1. After 24 hrs, proteins were extracted and subjected to Western blot analysis. The membrane was sequentially probed with the indicated antibodies. The results shown are representative of one of two independent experiments. **(H)** The IL-6 concentration of the cell culture medium was detected by ELISA. The data shown are representative of one of two independent experiments performed in triplicate. The results represent the mean ± SEM. Two-way analysis of ANOVA was used for comparison. ***P* < 0.01.
