# Supplementary figures and images for: JAK2V617F Mutation Promoted IL-6 Production and Glycolysis via Mediating PKM1 Stabilization in Macrophages
Source: Front Immunol. 2021 Feb 8;11:589048. doi: 10.3389/fimmu.2020.589048 (PMC7897702; doi:10.3389/fimmu.2020.589048)

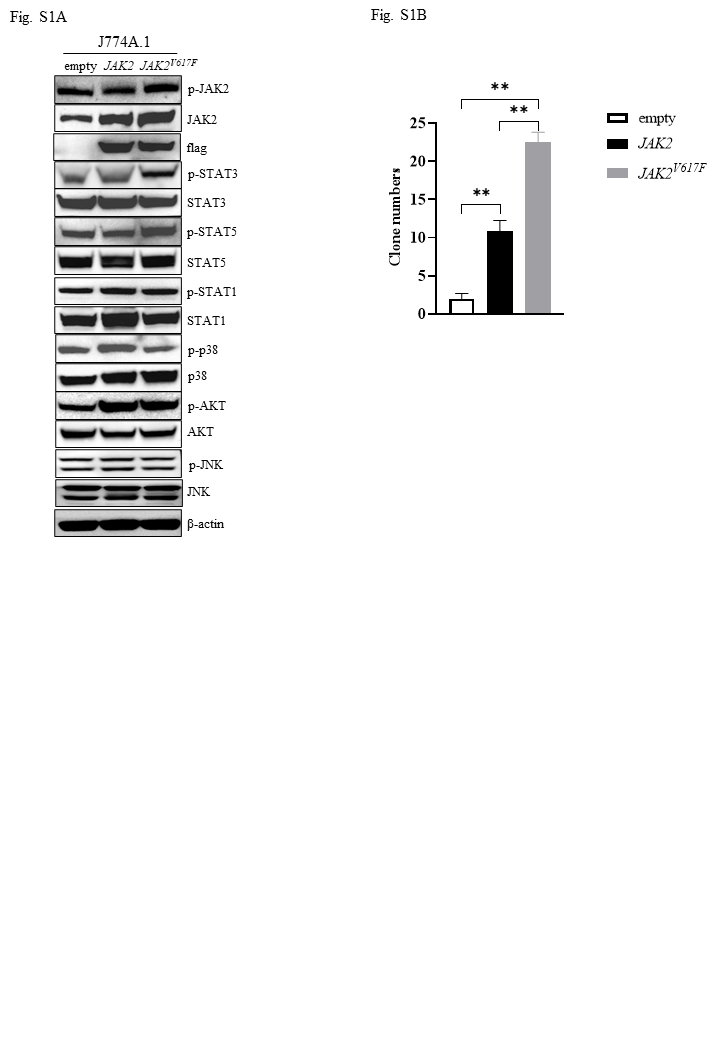

Supplement: Supplementary Figure 1 — Related to Figure 1 . (A) Western blot analysis. Proteins were extracted from the indicated cell lines and sub packaged equally for three groups (each group included the same amount of empty, JAK2 and JAK2V617F lysates), followed by separation on 8% SDS-PAGE gel. The membrane was sequentially probed with the indicated antibodies. The results shown are representative of one of two independent experiments. (B) Colony assay. The number of colonies was counted by ImageJ software after crystal violet staining. The data shown are representative of three independent experiments performed in duplicate. The results represent the mean ± SEM. One-way analysis of ANOVA was used for comparison. **P < 0.01; *P < 0.05; n.s., not significant. [file Image_1.tif]

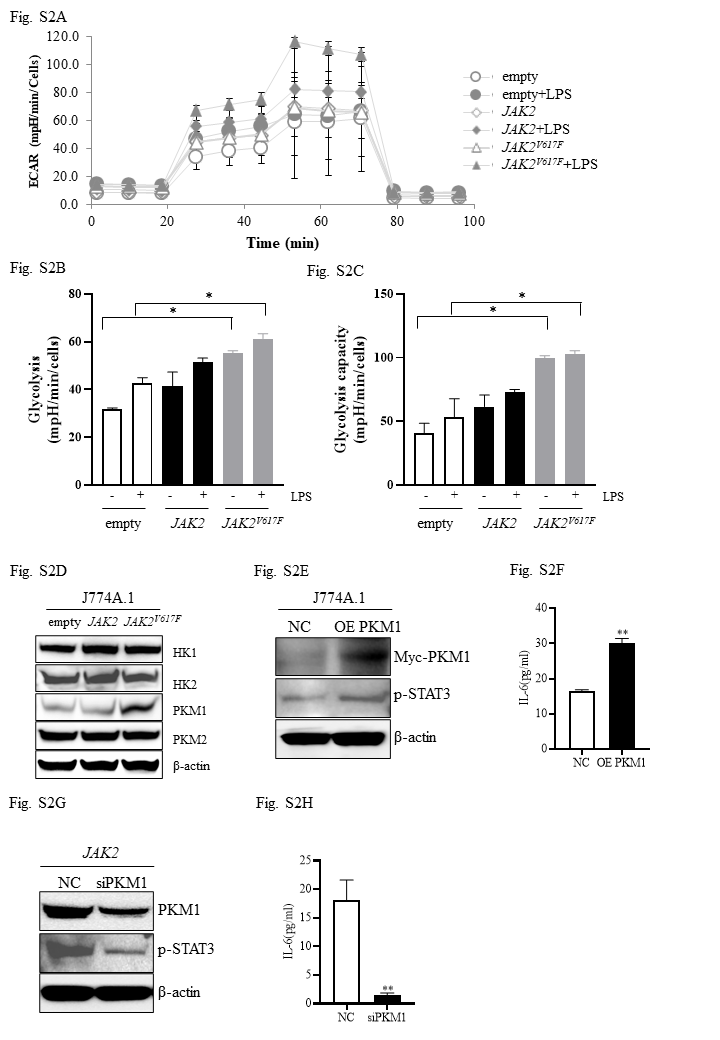

Supplement: Supplementary Figure 2 — Related to Figures 3 . (A) J774A.1 macrophages treated with or without LPS were seeded into wells, and the ECAR was determined by extracellular flux analysis. A representative plot of the ECAR over time of these cells with the addition of glucose (30 mM), oligomycin (2 mM), and 2-DG (50 mM), as indicated. (B) Glycolysis in the assay shown in Supplementary Figure 2A was quantified. The data shown are representative of one of two independent experiments. The results represent the mean ± SEM. Two-way analysis of ANOVA was used for comparison. *P < 0.05. (C) The glycolytic capacity in the assay shown in Figures 3A was quantified. The data shown are representative of one of two independent experiments. The results represent the mean ± SEM. Two-way analysis of ANOVA was used for comparison. *P < 0.05. (D) Proteins were extracted and subjected to Western blot analysis. The membrane was sequentially probed with the indicated antibodies. The results shown are representative of one of two independent experiments. (E) J774A.1 macrophages were transfected with empty or Myc-PKM1 expression vectors. After 24 hrs, proteins were extracted and subjected to Western blot analysis. The membrane was sequentially probed with the indicated antibodies. The results shown are representative of one of two independent experiments. (F) The IL-6 concentration of the cell culture medium was detected by ELISA. The data shown are representative of one of two independent experiments performed in triplicate. The results represent the mean ± SEM. Two-way analysis of ANOVA was used for comparison. **P < 0.01. (G) JAK2-expressing J774A.1 cells were transfected with a negative control siRNA or a siRNA against PKM1. After 24 hrs, proteins were extracted and subjected to Western blot analysis. The membrane was sequentially probed with the indicated antibodies. The results shown are representative of one of two independent experiments. (H) The IL-6 concentration of the cell culture medium was detected by EL [file Image_2.tif]
